# Quality control methods in musculoskeletal tissue engineering: from imaging to biosensors

**DOI:** 10.1038/s41413-021-00167-9

**Published:** 2021-10-27

**Authors:** Daniele Zuncheddu, Elena Della Bella, Andrea Schwab, Dalila Petta, Gaia Rocchitta, Silvia Generelli, Felix Kurth, Annapaola Parrilli, Sophie Verrier, Julietta V. Rau, Marco Fosca, Margherita Maioli, Pier Andrea Serra, Mauro Alini, Heinz Redl, Sibylle Grad, Valentina Basoli

**Affiliations:** 1 AO Research Institute Davos, Clavadelerstrasse 8, 7270 Davos, Switzerland; 2 Regenerative Medicine Technologies Lab, Ente Ospedaliero Cantonale (EOC), Via Tesserete 46, 6900 Lugano, Switzerland; 3 Department of Medical, Surgical and Experimental Sciences, University of Sassari, Viale San Pietro 43/b, 07100 Sassari, Italy; 4 Centre Suisse d’Electronique et de Microtechnique, Bahnhofstrasse 1, 7302 Landquart, Switzerland; 5 Center for X-ray Analytics, Empa - Swiss Federal Laboratories for Materials Science and Technology, Überlandstrasse 129, 8600 Dübendorf, Switzerland; 6 Istituto di Struttura della Materia, Consiglio Nazionale delle Ricerche (ISM-CNR), Via del Fosso del Cavaliere, 100 - 00133 Rome, Italy; 7 Sechenov First Moscow State Medical University, Trubetskaya 8, build. 2, 119991 Moscow, Russian Federation; 8 Department of Biomedical Sciences, University of Sassari, Viale San Pietro 43/b, 07100 Sassari, Italy; 9 Ludwig Boltzmann Institute for Experimental and Clinical Traumatology in AUVA trauma research center, Donaueschingenstraße 13, 1200 Vienna, Austria; 10 Austrian Cluster for Tissue Regeneration, Vienna, Austria

**Keywords:** Bone, Bone quality and biomechanics

## Abstract

Tissue engineering is rapidly progressing toward clinical application. In the musculoskeletal field, there has been an increasing necessity for bone and cartilage replacement. Despite the promising translational potential of tissue engineering approaches, careful attention should be given to the quality of developed constructs to increase the real applicability to patients. After a general introduction to musculoskeletal tissue engineering, this narrative review aims to offer an overview of methods, starting from classical techniques, such as gene expression analysis and histology, to less common methods, such as Raman spectroscopy, microcomputed tomography, and biosensors, that can be employed to assess the quality of constructs in terms of viability, morphology, or matrix deposition. A particular emphasis is given to standards and good practices (GXP), which can be applicable in different sectors. Moreover, a classification of the methods into destructive, noninvasive, or conservative based on the possible further development of a preimplant quality monitoring system is proposed. Biosensors in musculoskeletal tissue engineering have not yet been used but have been proposed as a novel technology that can be exploited with numerous advantages, including minimal invasiveness, making them suitable for the development of preimplant quality control systems.

## Introduction

Tissue engineering aims to study and develop new methods for the replacement of damaged or diseased tissue. The fundamental idea is to use the appropriate type of cells, seed them on suitable support materials, induce controlled cell differentiation by the addition of specific growth factors and use the mature construct for replacement of the lost tissue. Mesenchymal stromal cells (MSCs) are at the root of this technology due to their regenerative potential and capability to differentiate into osteogenic, chondrogenic and adipogenic lineages,^1^ which make them particularly attractive for musculoskeletal tissue engineering. To improve in vitro models and generate tissues more similar to native tissue, traditional two-dimensional (2D) cell culture methods have been translated toward establishing three-dimensional (3D) constructs. In the past 20 years, considerable progress has been made in moving tissue engineering^2^ closer to clinics, which opens the opportunity to treat several musculoskeletal diseases or injuries in the near future. Although research in this field has been rapidly growing, progress needs to be made toward translation from the bench to the bedside.^3^ The translation of a tissue engineered product, defined by the regulatory framework as an advanced therapy medicinal product (ATMP), includes standardization of protocols for the fabrication of the implants that meets good laboratory manufacturing and practice as well as regulatory aspects. Standardization and quality control are important aspects that can ensure a boost in clinical translation. These criteria require modern methods allowing for standardized quality control in a 3D setup. The development of standard protocols will help to ensure the reproducibility of experiments and quality in manufacturing products in the clinic and consequently can ensure safe translation to patients.

Each analytical technique has advantages and disadvantages and can be used at a specific stage during the development of engineered tissues. This review article aims to provide an overview of the main methods used for quality control in tissue engineering of the musculoskeletal system, with a focus on cartilage and bone regeneration, using emerging techniques in the field. The techniques described herein are divided into 3 groups depending on the possibility of a future implant of engineered tissues. According to this principle, destructive methods result in the samples being destroyed, so they cannot be used to perform further analyses or for clinical purposes. Examples of such methods include classical techniques such as gene expression analysis, immunohistological staining, and some imaging techniques. Nondestructive or noninvasive techniques maintain sample integrity; therefore, the same specimen can be used for further analyses with destructive techniques. However, the samples might not be suitable for clinical use, as the technique might affect cell behavior; for example, X-ray doses for microCT require extra attention for their mutagenic properties. Finally, conservative methods keep the sample intact and do not affect cell biology, ideally enabling use for clinical implantation. Some types of biosensors can be included in this group. However, there is still no reported use of biosensors in musculoskeletal tissue engineering. Therefore, the potential of sensors for quality monitoring is introduced by discussing meaningful studies from other fields, such as neurosciences, as biosensors may represent a potential tool for the advancement of tissue engineered products both in the preclinical and clinical settings. Furthermore, a selection of the main regulatory standards that can be applied in the field is discussed in the respective paragraphs. To conclude, standard procedures, together with innovative methods of quality control, can contribute to the identification of key factors that influence the safety and quality of products^4^ with the overall aim of quality control, thereby achieving full translational clinical potential.

## Methods for musculoskeletal tissue engineering

### Cell sources for musculoskeletal tissue engineering

The ultimate goal of tissue engineering is the restoration of the structure and function of tissues lost due to trauma or disease, including musculoskeletal tissues such as bone or cartilage. Despite the tremendous effort and the number of clinical and preclinical studies in diverse fields, there is still a paucity of clinically approved solutions including cells with or without materials.^3,5^ A successful tissue engineering approach is complex, with many factors to be tuned to recreate functional tissue that can integrate with the native environment. Among these factors, the cells used represent the building block, and their careful selection and management is of critical importance. For a recent overview, see the TERMIS book series (https://www.springer.com/series/13441). For more than a decade, mesenchymal stromal cells have been indicated as the most promising source in regenerative medicine/tissue engineering of the musculoskeletal system because of their regenerative potential and their physiological tendency to differentiate toward cells of the mesodermal lineage, such as osteoblasts and chondrocytes.^6^

There are several active clinical trials involving MSCs (clinicaltrials.gov, *n* = 237 actively recruiting studies as of August 2020), with more than 50 studies focused on the treatment of musculoskeletal disorders and osteoarthritis *in primis*. Bone marrow, adipose tissue and umbilical cords are the major sources of stem cells in current trials, probably owing to tissue accessibility, the number of cells that can be obtained and the large number of preclinical studies conducted. Even though biobanking of bone marrow-derived MSCs is possible, they have mainly been studied for autologous use in musculoskeletal applications^7^. Analogous considerations can be made for adipose tissue-derived stromal cells (ADSCs) and stromal vascular fractions (SVFs).

Umbilical cord-derived stem cells are promising, especially for allogeneic use, since cord blood or Wharton Jelly can be collected from term births with no invasive procedure.^8^ Allogeneic transplantation relies on the use of biobanks, with higher requirements in terms of manipulation and regulations (see^9–12^ for reference on production and cryopreservation of cells).

Induced stem cells are a highly promising cell type in the field of regenerative medicine. In 2006, Takahashi and Yamanaka, by screening 24 different genes, concluded that adult fibroblasts could be reversed to totipotent cells by upregulating Oct3/4, Sox2, c‐Myc, and Klf4. These reprogrammed cells, designated as induced pluripotent stem cells (iPSCs), displayed a typical embryonic stem (ES) cell‐like morphology and growth physiology.^13^

On the other hand, the use of cells with a more mature phenotype (i.e., differentiated cells) has been more commonly employed for cartilage tissue engineering than for bone. Indeed, autologous chondrocyte implantation (ACI) has already been used in clinical practice, although with variable results,^14^ donor site morbidity and cell dedifferentiation during expansion and after implantation. Cells for ACI can be derived from low-weight bearing areas of the joint,^15,16^ auricular cartilage^17^ or nasal chondrocytes,^18^ the latter showing promising results for the development of hyaline cartilage. For bone tissue engineering applications, rather than mature cells, potential, autologous bone grafts are usually employed with good results and still represent the gold standard in the treatment of large bone defects.^19,20^

### Regulatory standards for cell isolation and manipulation

Interest in MSCs for the treatment of several diseases has constantly increased in recent years due to their ability to improve tissue regeneration in patients who are refractory to other therapies. Minimal criteria for the identification and characterization have been proposed for both MSCs^1^ and ADSCs.^21^

Most of these cell preparations are autologous; thus, the cells have to be isolated, expanded, and manipulated before being reinjected into the patient. In November 2017, the European Commission published a set of new guidelines on good manufacturing practices (GMPs) specific to ATMPs. The guidelines for GMP 2003/94/EC6 highlighted three important pillars for patient and product standardization: (1) Standardization of quality control methods; (2) Integration of procedures for product tracking; and (3) Establishment of self-inspection analysis to monitor quality standards. Later, these principles became an integral part of legislative redlines concerning TE products 2004/23/EC and 2006/17/EC. Furthermore, ATMPs have mainly focused on the establishment of regulations for somatic cell therapy and gene therapy medicinal products (European Directive 2001/83/EC), which regulate the use of standard operating procedures (SOPs), guidelines, reference manuals, donor records, and reporting forms for tissue or cell quality for their quality management systems (QMSs)^22^.

All stages of cell processing and storage must comply with strict GMP guidelines and must be accompanied by accurate documentation.^23,24^ These guidelines define general measures to ensure that the processes required for production and validation are clearly defined, validated, reviewed and documented and that personnel, premises and materials are suitable for the production of pharmaceutical and biological products.^25^ The quality and safety of the product must be guaranteed, from the first phases of production, to minimize microbiological and cross-contamination through continuous environmental monitoring^26^. The first World Health Organization (WHO) draft text on GMPs was adopted in 1968. A Supplementary Annex on Biological Standardization (ECBS) of biological medicinal products was adopted in 1991, setting out the general approach to quality control of biological medicinal products such as vaccines, blood and blood products, antigens, cell and tissue therapies, biopharmaceuticals and others.^27^ Worldwide, more than 100 countries have incorporated WHO GMP requirements into their national medicine laws. Grade A facilities are essential for production and packaging of the product under aseptic conditions, which are normalized by specific quality controls to produce a safe ATMP for direct human application. The International Standards Organization (ISO) provides requirements, specifications or guidelines that can be used to ensure that materials, products, processes and services are safe, efficient and environmentally friendly.^28^ Table 1 summarizes applicable standards and relevant GMP literature for cell isolation and manipulation.

**Table 1:** Standards and GMP literature applicable to cell manufacturing

Standard code/Reference	Standard title/description	Standard status	Standard development stage
ISO 20387:2018	Biotechnology—General requirements for biobanking	Published	–
ISO/WD TS 22859	Biotechnology—Requirements for human mesenchymal stem and stromal cells derived from umbilical cord	Under development	Stage 20.20 (Working draft study initiated)
ISO/AWI 24651	Biotechnology—Requirements for human mesenchymal stromal cells derived from bone marrow	Under development	Stage 10.99 (new project approved)
^9–11^	GMP and regulatory elements for MSC production and banking	–	–
^251^	GMP for iPS	–	–
^26^	Cleanroom ISO and other regulations	–	–

### Development of 3D constructs and 3D bioprinting

Tissues of the musculoskeletal system are characterized by their complex 3D environment and hierarchical nature of the matrix component on the microscale, such as the proteoglycan gradient^29,30^ and collagen fiber orientation in articular cartilage.^31,32^ To develop advanced biologically functional in vitro models, special emphasis is placed on 3D culture setups to better mimic the 3D environment in the target tissue. Among additive manufacturing techniques, 3D bioprinting is the most prominent approach to fabricate patient-specific and thus anatomically shaped 3D biological functional scaffolds combining cells, supporting biomaterial and bioactive molecules for implantation, drug screening or in vitro models.^33^

The ultimate aim of biofabrication technologies is to produce patient-specific tissues or anatomically shaped organs by using autologous or allogeneic cells combined with a biomaterial to replicate the original geometry of the diseased or damaged tissue.^34,35^ There are promising results in bioprinting implants for bone repair^36–40^ or complex multidimensional structures to produce (cell-free) scaffolds mimicking the osteochondral environment for full cartilage defects.^41–47^ However, major limitations of these studies are the missing tools to characterize the printing quality and reproducibility postprinting and to investigate cell viability, process-induced cell stress, senescence, apoptosis or related cell phenotypic changes within the 3D constructs.^48^ In the literature, some basic experimental works identifying cell death induced by the 3D printing process^49,50^ and different behaviors of cell differentiation comparing casted vs. printed constructs^51,52^ have been published. This topic needs to be studied more in-depth to better understand the impact of the printing process on cells and evaluate whether printed constructs outweigh simple casted implants with regard to their biological performance. In addition to biological evaluation, functional characterization, such as histological or mechanical characterization, of printed engineered constructs is essential. For example, mechanical tests of both cartilage-like and bone-like constructs are needed to quantify their mechanical strength, elasticity and absorption at the material interface and therefore to understand their interaction with the tissues.^53^ However, while the mechanical characterization of bone has been well established for preclinical studies, functional testing of cartilage has yet to be fully characterized.^54^ In fact, although it is well known that the mechanical properties of engineered cartilage constructs have to match those of native cartilage at load-bearing joints, there is still a lack of standard procedures for testing the mechanical properties of engineered articular cartilage, which often leads to an inability to compare the results of different studies. A recent review by Patel and colleagues^55^ discusses mechanical testing of articular cartilage and underlines that compression testing (various modes such as ramp, stress relaxation, creep, dynamic and testing configurations such as unconfined, confined, in situ) is the most common test performed, followed by an evaluation of frictional properties. Similarly, a systematic review published by Marchiori et al.^56^ discussed cartilage mechanical characterization, showing that unconfined compression (both dynamic and static conditions) is the most diffuse configuration, although it does not have a standard reference. Additionally, confined compression tests are more common and standardized than indentation tests, probably due to the more straightforward experimental and analytical configuration. Mechanical tests of cartilage devices are not reported by ISO, whereas the FDA has clear guidelines for these devices,^57^ specifically for knees, where they recommend the major evaluations that have to be carried out, such as the ability of an implant to withstand expected in vivo static and dynamic loading (e.g., compression, shear and tension) and the most relevant measurements, such as maximum recoverable compressive strain, aggregate modulus, shear modulus, permeability and complex shear modulus G* measurements.

Furthermore, more specifically for 3D printed constructs, GLPs that follow quality criteria and standards for characterization of the printed samples need to be defined to bring the field closer toward clinical translation and application.

To date, there are no regulatory guidelines of the US FDA or other institutions available for 3D bioprinting cellular or acellular constructs for application in clinical studies, which can be explained by the infancy of the technologies. In contrast to living 3D bioprinted products, nonliving and thus cell-free printed devices or surgical tools made of medical-grade plastics (e.g., polyether ketone), which are tolerant for subsequent sterilization procedures,^58^ are already in clinical use. Medical applications of these products include the use of anatomical models based on patient radiological data serving as intraoperative surgical guides, biomodels to plan surgical approaches or education tools^59–63^ and constructs serving as space fillers or bridges for dental use.^64^ To produce the abovementioned biomedical products, the FDA released guidance covering device design, software workflow, material controls, postprocessing and process validation (Technical Considerations for Additive Manufactured Medical Devices Guidance for Industry and Food and Drug Administration Staff, FDA-2016-D-1210). ISO provides a similar document, the ISO/ASTM 52900:2015 (ASTM F2792) Additive manufacturing—General principles—Terminology (2015).

## Quality monitoring of tissue engineering constructs

### Destructive methods for quality analysis

#### Analysis of cellular toxicity

Cytotoxicity testing is a first step toward ensuring the biocompatibility of a biomaterial that might be used as a medical device. Cytotoxicity testing is reported in the ISO 10993-5: “Tests for Cytotoxicity—In Vitro Methods” This ISO standard describes a set of rapid, sensitive, and inexpensive tests designed to standardize cellular toxicity and quantify the effect of biologically harmful products. These tests are widely used for the assessment of cell viability in 3D tissue engineered constructs.

It is crucial to follow specific assay conditions. Details on the procedures in the preparation of samples are reported in part ISO 10993-12:2012(E). For example, in the extract test method, extracting conditions should simulate the clinical use conditions without altering the chemical properties of the samples.

According to ISO 10993, a reduction in cell viability by more than 30% is considered to be a cytotoxic effect. This cellular toxicity can be determined following different evaluation categories: assessment of cell damage by morphological means, measurement of cell growth, and measurement of specific aspects of cellular metabolism. Additional qualitative morphological scoring is available to assess changes in the general morphology, vacuolization, detachment, cell lysis and membrane integrity.

One of the most common cytotoxicity tests is the (3-(4,5-dimethylthiazol-2-yl)-2,5-diphenyltetrazoliumbromid) (MTT) assay (ISO 10993, Annex C), based on an optical density measurement.

**Fig. 1:**
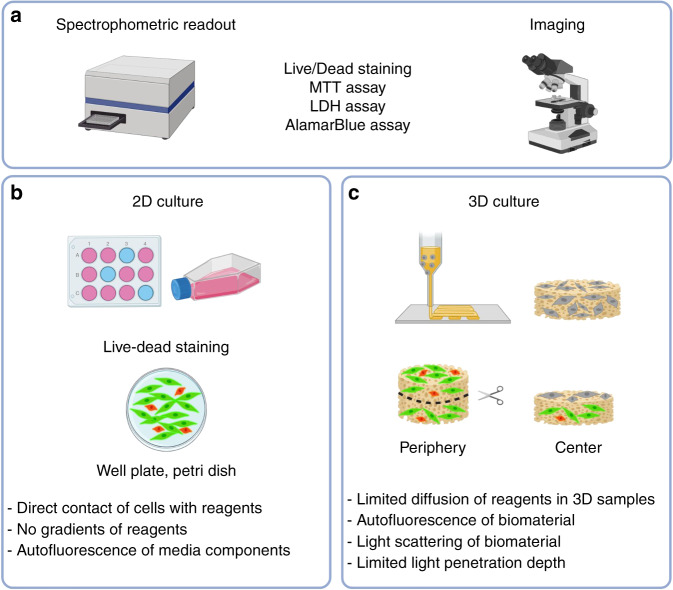
Analysis of cell viability in 2D and 3D cultures. **a** Comparison of viability and metabolic activity assessment via spectrophotometric readouts or (confocal) microscopy in 2D and 3D in vitro culture setups. **b** 2D cultures are shown as monolayers in well plates or tissue culture flasks where reagents have direct contact with the cells. **c** 3D samples are characterized by the presence of a biomaterial as a supportive structure (e.g., fabricated via 3D bioprinting) in which the cells are embedded. Reagents need to diffuse through the material to stain or be metabolized by the cells, and thus, a limitation in penetration depth may result in low or no positive staining toward the center of the 3D construct, as illustrated for live-dead staining. Furthermore, many biomaterials or compounds present in the materials can interfere and show an autofluorescent signal that, in the worst case, results in higher readout values or background staining than that derived from the cells only

In addition to the evaluation of the cytotoxicity of the compounds connected to ISO 10993, there are various other methodologies to monitor cell viability and metabolic activity in 2D and 3D culture setups, including live-dead staining and lactate dehydrogenase (LDH) assays (Fig. 1).

To date, the abovementioned methodologies, based on spectrophotometric or imaged-based readouts, have been established and are routinely used in 2D cultures where the reagents are applied on cell monolayers and are thus in direct contact with the cells. The main difference between 2D and 3D cultures is that in 3D cultures, the cells are embedded within a biomaterial or seeded onto a 3D scaffold. Reagent-based assays to assess cell viability or differentiation have some limitations when cells are embedded in a 3D material: on the one hand, materials may absorb dyes due to electrostatic interactions and thus interfere with subsequent absorbance or fluorescence measurements. Furthermore, many biomaterials or compounds present in the materials interfere with an autofluorescent signal that, in the worst case, result in higher readout values or background staining compared to the signal derived from the cells.

Another issue addresses the diffusion of the reagents into the 3D construct to assess the viability or metabolic activity of the full construct. In 3D cultures, the reagents need to diffuse through the material to stain the cells or be metabolized by the cells. The presence of a biomaterial reduces the diffusion of the reagents, and there is the risk of a penetration depth limit. Thus, gradients with high concentrations will occur at the periphery of the large, centimeter-scale samples with decreasing concentrations toward the center unless the incubation times are optimized to the specific biomaterials and geometry (shape and size).^65^

#### Analysis of molecular markers

The analysis of gene expression is a powerful tool for the evaluation of tissue engineering constructs since it provides information about the differentiation status and maturation of a construct, allows control of the status of cell differentiation pathways, and enables monitoring of the effectiveness of drug treatments.

**Fig. 2:**
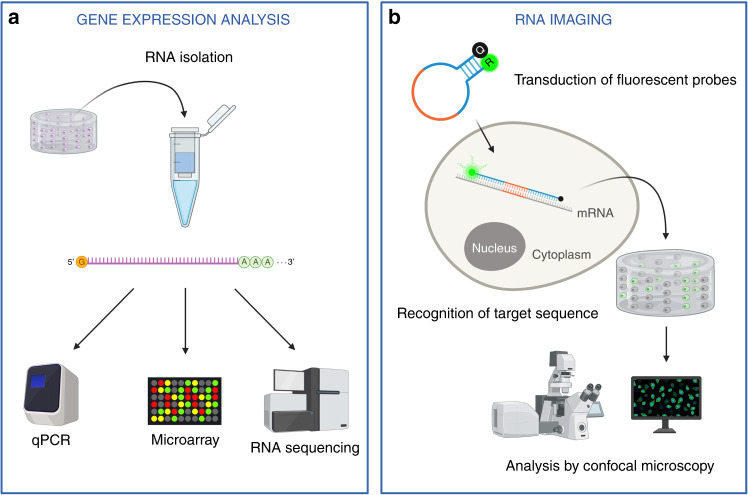
Quality monitoring via gene expression analysis. **a** Gene expression analysis starts with RNA isolation, which implies the destruction of the sample. Different techniques can then be employed to assess gene expression levels, including qPCR analysis, microarrays, and RNA sequencing. **b** Intracellular RNA can be imaged using fluorescent nucleic acid-based probes. The probe should be transfected into the cells; once inside, the probe anneals to the target sequence, leading to an increase in fluorescence, which can then be analyzed via confocal microscopy

There are different techniques that can be used for the analysis of gene expression (as schematically depicted in Fig. 2), each with specific advantages and disadvantages. Among those, the most useful in the field of tissue engineering are quantitative real-time polymerase chain reaction (qPCR), probe-based microarrays, RNA sequencing (RNAseq), or RNA imaging of fixed or live cells using specific probes.

qPCR-based approaches represent the method of choice for targeted gene expression analysis. The main pros of qPCR are specificity, sensitivity, customizability, flexibility, robustness, constant technology and reagent development, and availability of validated assays to cover the whole human transcriptome and a large number of noncoding (nc)RNAs. The main disadvantage of qPCR is that it is a destructive technique, and even though a good number of targets can be analyzed in the same sample, it does not allow for a transcriptome-wide analysis and requires prior knowledge of targets to be investigated.

A different approach is based on gene expression microarrays that can analyze thousands of genes in the same sample. The advantage of microarray technology over qPCR is undoubtedly the possibility of analyzing a larger number of targets in the same sample, on the order of thousands of genes, therefore covering a larger portion of the transcriptome and allowing for pathway analysis.^66^ The main disadvantage is that microarrays are probe-based, thus requiring prior knowledge of the sequences to be analyzed.^67^

Some of these limitations can be overcome by RNAseq methods. The main advantage of RNAseq is that its approach is unbiased and allows for a transcriptome-wide analysis. RNAseq disadvantages are mainly destructivity and high costs, although they decrease with time.^68,69^ Moreover, the analysis of data is complex and requires specialized bioinformatics skills.

More recently, imaging techniques based on RNA probe hybridization have been developed. These techniques are less commonly used in the field of musculoskeletal tissue engineering. RNA probe-based imaging has the great advantage of being potentially adapted for live cell imaging, as described by some authors with molecular beacons.^70–73^ Its main limitation is the low multiplexing capability and a high level of optimization required for signal detection and maintenance of cell viability, especially in 3D constructs.

Regarding quality guidelines, there are a few documents applicable to gene expression analysis, but standards are under development for sequencing. Table 2 summarizes standards and literature on quality relevant for gene expression analysis.

**Table 2:** ISO standards and literature applicable for gene expression analysis

Standard code/Reference	Standard title/description	Standard status (2020)
ISO 16578:2013	Molecular biomarker analysis—General definitions and requirements for microarray detection of specific nucleic acid sequences	Published
ISO 20395:2019	Biotechnology—Requirements for evaluating the performance of quantification methods for nucleic acid target sequences—qPCR and dPCR	Published
ISO/AWI 20397-1	Biotechnology—General Requirements for Massive Parallel Sequencing - Part 1: Nucleic acid and library preparation	Under development
ISO/DIS 20397-2	General requirements for massively parallel sequencing—Part 2: Methods to evaluate the quality of sequencing data	Under development
^252^	Quality Assurance of RNA Expression Profiling in Clinical Laboratories	

#### Histological assays

One of the main purposes in tissue engineering is to create organized and functional tissues that are as close as possible to physiological tissues.^74^ Therefore, a morphological assessment is necessary to evaluate the quality of the in vitro matured tissues through histological and histochemical techniques.^75^

One of the principal tissue stains used in histology is hematoxylin-eosin staining (H&E), which gives a general overview of cell distribution and tissue organization. However, this type of assay does not provide much information regarding the quality of the extracellular matrix produced, i.e., of the presence of proteoglycans and of cartilage-specific collagens. For this purpose, extracellular matrix analysis is commonly performed by staining with Safranin-O,^76^ a dye that highlights the presence of proteoglycans and glycosaminoglycans. Counterstaining is usually achieved with Fast Green and Weigert’s hematoxylin for cell nuclei. The staining is characterized by a red appearance in the presence of negatively charged glycans, with a green color indicating other cells and collagen. Other types of staining, such as Masson’s trichrome, are used for the visualization of collagenous connective tissue fibers in tissue sections.^77^ Tolonium chloride (also known as toluidine blue) and Alcian blue staining can indicate the presence of acidic polysaccharides such as glycosaminoglycans in cartilage and other body structures.^78^ Another method is to use dimethyl methylene blue (DMB or DMMB) for cartilage detection.^79^ These techniques, although qualitative, do not allow us to discriminate which specific molecule is expressed in the construct. Especially during stem cell commitment toward chondrocytes, it is extremely necessary to understand the quality and stage of differentiation. The main protein markers associated with cartilage are type II collagen, which constitutes the articular and hyaline cartilage; type I collagen, conventionally associated with fibrous cartilage; and type X collagen and matrix metallopeptidases (MMPs), usually expressed during hypertrophic differentiation, when cells commit to osteochondral and bone formation. The expression and distribution of those proteins can be analyzed by immunohistochemical staining.

In light of bone tissue engineering, the main characteristic is the production and thus presence of a mineralized matrix, which can be analyzed in different ways. Both Alizarin Red and Von Kossa staining can be performed on histological sections and in monolayer cultures to visualize deposited bone-like matrix. Other methods to stain calcium can include fluorochrome labels, such as calcein green, xylenol orange or tetracyclines, although their use is more applied to assess new bone formation in in vivo studies.^80^ Other fluorescent dyes with the ability to bind to hydroxyapatite (HA) have been developed and can be used in cell cultures and tissue sections, as in the case of Giemsa^81^ or fluorescein-bisphosphonate conjugates.^82^ In addition to mineralization, immunohistochemical detection of bone-specific proteins can be of course envisaged to assess the quality of bone.^83,84^

Although a first analysis through histological techniques can provide an idea about the morphology of the construct and its quality, this approach still has some limitations. The main advantage of using classical histological staining is its low cost, direct visualization where tissue-specific extracellular matrix is deposited, and high throughput. However, the qualitative evaluation of specimens should be performed by immunohistochemical assays in which specific antibodies bind proteins of interest, allowing effective assessment. Although this type of technique appears simple, it can be affected by several variables, including temperature, antibody concentration, fixation, demasking protocols, and binding to biomaterials, which can easily lead to false-positive or negative results. Thus, the parallel staining of internal and external controls, e.g., native tissue specimens or nondifferentiated cells, is of great importance for the evaluation of the staining results.

The main limitations of this technique already arise from the fixing method, which can radically change the recognition of the antibody used. The inclusion phase represents another limiting step, which can totally change the interpretation of the result; indeed, paraffin or resin embedding can generate artifacts in the results by modifying 3D constructs if mainly constituted by hydrogels.^85,86^ However, the use of snap freezing and subsequent cutting with cryotome could partly overcome this limit. Finally, the extracellular matrix can often interfere with the detection of fluorescent antibodies because of its intrinsic autofluorescence, which could easily lead to a false interpretation. Furthermore, histological and immunohistological analyses involve the destruction of the sample and the use of expensive reagents such as antibodies.

#### Laser confocal microscopy

**Fig. 3:**
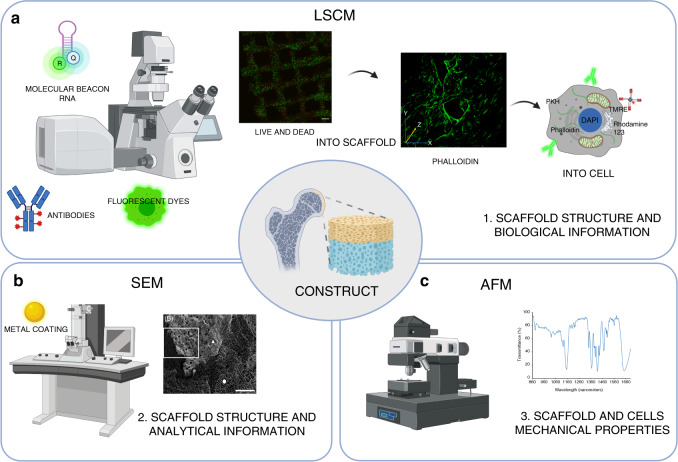
Summary of microscopy techniques for qualitative and quantitative assessment of cells, scaffolds and tissue engineered constructs. **a** With laser scanning confocal microscopy (LSCM), it is possible to analyze the expression of specific proteins and nucleic acids and to assess the biological responses and morphological organization of cells using fluorescent dyes. **b** With scanning electron microscopy (SEM), it is possible to gather high-resolution structural and analytical information about tissues and constructs. **c** Atomic force microscopy (AFM) allows us to analyze the surface and mechanical properties of constructs

Laser confocal scanning microscopy (LSCM) (Fig. 3) has become an important instrument that has been widely used in materials science,^87^ cytobiology, morphology,^88^ pharmacology,^89^ neuroscience^90^ and tissue engineering.^91,92^

LSCM can acquire fluorescence from only one focal point at a time, allowing the generation of high-quality images. Compared with a traditional optical microscope, a confocal microscope can obtain extremely precise 3D images and measure subcellular structure.^93^

Similar to classical histology, cell characteristics, cell organization within a scaffold, and production of extracellular matrix proteins can be analyzed by using specific fluorescently labeled antibodies or other molecules that target cellular structures such as nucleic acids, membranes, and mitochondria (Table 3).

**Table 3:** Overview of fluorescent staining methods to target specific cellular components

Target	Type of labeling	Protein or nucleic acid specific binding
Protein of interest	Antibody labeling (fluorochrome conjugation on primary or secondary antibody)	Yes
Membrane	Lipophilic dyes such as PKH dyes: - PKH2 (green) - PKH26 (red) - PKH67 (green)	No
Cytoskeletal components	Phalloidin conjugates (actin) Paclitaxel or docetaxel conjugates, other Taxol derivatives (tubulin)	Yes
Organelles	Rhodamine 123 (mitochondria) TMRE, TMRM (mitochondria, endoplasmic reticulum) LysoTracker (lysosomes)	No
Nucleic acid	DAPI, Hoechst dyes (dsDNA, A-T rich regions) EthD-1, EtBr (dsDNA) SYBR dyes (dsDNA minor grove region) Oligonucleotide probes (RNA, ssDNA, dsDNA)	Yes (structure or sequence dependent binding)

*DAPI* 4′,6-diamidino-2-phenylindole, *EtBr* ethidium bromide, *EthD-1* ethidium homodimer-1, *PKH* Paul Karl Horan (note: PKH dyes are named after their discoverer), *TMRE* tetramethylrhodamine ethyl ester, *TMRM* tetramethylrhodamine methyl ester.

Confocal microscopy can be used both for semiquantitative studies^94–96^ and for qualitative evaluation of samples, with the possibility of reconstructing the 3D organization.^95^

Labeling of cells can be performed in fixed or living constructs, depending on the outcome needed, and quality assessments can be performed with different methods. The tracking of cells in a tissue engineering study is of paramount importance; for this reason, common techniques aim to label cell components with or without the use of antibodies.

Frequently, transfection of cells by using genes that encode fluorescent proteins, such as eGFP, tdTomato, or mCherry, is used to study cell morphology and organization; the use of vital dyes that label membranes is of considerable interest for the study of cell migration inside 3D tissues or in scaffolds. PKH dyes are used for monitoring cell trafficking and function, allowing the analysis of live tissues over time.^97,98^ This method is usually stable for some cellular doublings, but the signal can be diluted or lost in long-term experiments with high cell proliferation, leading to a decrease in quality or discontinuation of the analysis. Labeling of cytoskeletal components with phalloidin or Taxol derivative conjugates allows the visualization of fixed cells without the possibility of longitudinal application.

The labeling of DNA by DAPI, Hoechst dyes, or ethidium homodimers can facilitate the identification of cells into constructs, and higher concentrations of dyes can also penetrate and stain nuclei in live, intact cells.

Other molecules such as tetramethylrhodamine ethyl ester (TMRE) and tetramethylrhodamine methyl ester (TMRM) are membrane potential-sensitive, cationic fluorophores^99^ that stain mitochondria for oxidative stress and damage.

The study of cell viability, proliferation, and adhesion into the scaffold can also be performed using different methods. One possible approach uses phosphor dots inserted into the scaffold that become imperceptible when cells grow over them.^100^ Calcein-AM and ethidium homodimers are used for live/dead staining, allowing the study of cell viability in the construct. Nonfluorescent calcein-AM freely enters live cells where the acetoxymethyl ester is removed by hydrolysis by intracellular esterases; low concentrations of ethidium homodimer can cross the cell membrane, only in case of cell damage or death, and can bind to the DNA minor grove.^101^ The main advantage of using the live/dead method in confocal microscopy is the opportunity to evaluate cell viability in a 3D setting. However, one of the fundamental limitations of this technique is that it is necessary to acquire information in a short time once the sample has been stained, as calcein becomes toxic and can easily give false results. Furthermore, it is no longer possible to use and culture the constructs after staining.

Recently, a new method for the quantification of specific mRNA in living cells has been proposed with molecular beacons.^70–73^ These are hairpin-shaped nucleic acids, with a fluorescent reporter in one end and quencher on the other. Once the molecular beacon recognizes and anneals to the cytoplasmic target RNA, the structure opens, allowing the fluorophore to emit fluorescence. This conformation allows the real-time detection of the presence of a specific RNA target, making semiquantification possible.

The main limitation in LSCM depends on the optics, where a z-stack acquisition of 200 µm can restrict deeper analysis. Nonetheless, this method can be used for the observation of living cells in a complex 3D environment. Sample preparation can involve the use of antibodies and dedicated optical supports that could be quite expensive on a large scale. The use of dyes for scanning acquisition can interfere with further culturing and use. However, the broad use of confocal microscopy has some disadvantages. For example, the use of powerful lasers on living cells over a long period of time can create certain phototoxicity that can damage samples, while for fixed samples, photobleaching can wreck the fluorescent signal. However, the introduction of two-photon LSCM, where two low-energy photons enable stimulation of a fluorochrome instead of a single strong photon, partially solved these problems, reducing phototoxicity and photobleaching in samples.^102^

Regarding the guidelines proposed by the International Organization for Standardization, ISO 21073:2019 is applied only to confocal single point scanners using single photon excitation procedures, and this document referred to more general documents as ISO 8039, ISO 10934-1, and ISO 10934-2 (Table 4).

**Table 4:** List of standards applicable to different microscopy techniques and sample preparations

Standard code/Reference	Standard title/description	Field of application
ISO 21073:2019	Microscopes—Confocal microscopes—Optical data of fluorescence confocal microscopes for biological imaging	Confocal microscopy
ISO 18337:2015	Surface chemical analysis—Surface characterization—Measurement of the lateral resolution of a confocal fluorescence microscope	Confocal microscopy
ISO 25178-607:2019	Geometrical product specifications (GPS)—Surface texture: Areal—Part 607: Nominal characteristics of noncontact (confocal microscopy) instruments	Confocal microscopy
ISO 8039:2014	Microscopes—Values, tolerances and symbols for magnification	Light microscopy
ISO 10934-1:2002	Optics and optical instruments—Vocabulary for microscopy—Part 1: Light microscopy	Light microscopy
ISO 10934-2:2007	Optics and optical instruments—Vocabulary for microscopy—Part 2: Advanced techniques in light microscopy	Light microscopy
ISO 11039:2012	Surface chemical analysis—Scanning probe microscopy—Measurement of drift rates	Scanning probe microscopy
ISO 11775:2015	Surface chemical analysis—Scanning probe microscopy—Determination of cantilever normal spring constants	Scanning probe microscopy
ISO 18115-2:2013	Surface chemical analysis—Vocabulary—Part 2: Terms used in scanning probe microscopy	Scanning probe microscopy
ISO 11952:2019	Surface chemical analysis—Scanning probe microscopy – Determination of geometric quantities using SPM: Calibration of measuring systems	Scanning probe microscopy
ISO 27911:2011	Surface chemical analysis—Scanning probe microscopy—Definition and calibration of the lateral resolution of a near-field optical microscope	Scanning probe microscopy
ISO 13095:2014	Surface chemical analysis—Atomic force microscopy—Procedure for in situ characterization of AFM probe shank profile used for nanostructure measurements	Atomic force microscopy
ISO 21222:2020	Surface chemical analysis—Scanning probe microscopy—Procedure for the determination of elastic moduli for compliant materials using atomic force microscope and the two-point JKR method	Scanning probe microscopy
ISO 17025	Testing and calibration laboratories	General
ISO 13322-1:2014	Particle size analysis—Image analysis methods—Part 1: Static image analysis methods	image analysis
ISO 14887:2000	Sample preparation—Dispersing procedures for powders in liquids	Sample preparation
ISO 14488:2007	Particulate materials—Sampling and sample splitting for the determination of particulate properties	Sample preparation

ISO 21073:2019 can be applied to LSCM to image fluorescent biological specimens with high performance, increasing the repeatability, reliability, consistency, and overall quality of output. ISO 21073:2019 includes mechanisms for the resolution and strength of optical sectioning, uniformity of field and centering accuracy, coregistration accuracy, stability of illumination power, field number of the confocal scan optic and scanning frequency. In addition, ISO 21073:2019 includes other information regarding the specific wavelength of light required to excite a fluorescent molecule, such as a fluorescent antibody or fluorescent protein, the emission of light at emission and wavelength range of light collected by the photodetector, the pinhole diameter in terms of the low numerical aperture approximation, the ratio of signal to noise, and the glycerol air and water immersion refractive index.

#### Light-sheet microscopy

In addition to conventional high-resolution microscopy, optical imaging solutions exist that allow for the label-based and label-free analyses of small to larger tissues (cm-range). Light-sheet microscopy (LSM) is essentially a nondestructive microtome that provides 3D images of tissue constructs. While it is still based on the detection of fluorescent labels, it is also typically referred to as light-sheet fluorescence microscopy (LSFM). Although LSFM is not label-free, it has advantages over conventional microscopy and even conventional histology/pathology, especially in rapid volumetric microscopy; that is, it serves end-point analyses as well as monitors live tissue at high-resolution and thick tissue constructs.^103,104^ Images are obtained by illuminating a focal plane at an angle of 45° relative to the vertical direction, and the fluorescent signal is imaged orthogonally to the incoming light. LSFM can easily reach intracellular resolutions of <1.5 µm and extreme tissue penetration depths of >200 µm.^105,106^ The disadvantages of LSFM for the nondestructive monitoring of tissue samples are label-dependent detection and a limited penetration depth. Furthermore, this method can be used to image only transparent samples but not calcified tissue, such as bone.^107^

#### Scanning electron microscopy

Scanning electron microscopy (SEM) uses high-energy electron beams to image the surface of samples and provides information about sample morphology, chemical composition, and crystalline structure. The signals produced by the interaction between accelerated electrons and the sample comprise secondary electrons and backscattered electrons used for sample imaging. X-rays, specifically emitted by each element, are used to determine chemical compositions. In particular, secondary electrons have very low energies on the order of 50 *eV*, so imaging is limited to the superficial nanometers of the sample, whereas backscattered electrons are reflected from deeper regions (<100 nm) of the sample by elastic scattering. The spatial resolution of SEM ranges between 1 nm and 20 nm, depending on the size of the electron spot, which in turn depends on both the wavelength of the electrons and the electron-optical system producing the scanning electron beam.

SEM has been mainly employed for materials characterization, e.g., nanoparticle characterization or surface topography;^108,109^ however, it has demonstrated great potential in imaging biological samples, such as tissue fragments and cells.^110^

To image a biological sample with a conventional SEM, vacuum is required, and therefore, the sample must be dried, frozen and coated with a metallic layer. The development of the environmental SEM (ESEM) in the late ‘80 s has allowed the analysis of samples containing water or other volatile substances because of differential pressure-limiting apertures in combination with a pumping system in the path of the electron beam that maintain the gaseous environment around the sample. However, the electron gun itself is kept at standard pressures (10^–6^ to 10^–7^ *torr)*.

One application that involves the use of SEM is immunocytochemistry on biological samples.^111,112^ The antigen/antibody complex is labeled with a probe that produces a high secondary or backscattered electron signal. Colloidal gold probes are the selected probes due to their low background, difference from biological tissue components, and distinctive X-ray signal. Controls should be carefully chosen to check the performance of individual reagents or sample contaminations: a known positive control avoids false negatives and tests the effectiveness of the labeling procedure (reactivity of antigen, antibody, and markers), whereas a negative control assesses methodological nonspecificity of the technique, such as the secondary antibody labeling capacity (incubation with the secondary antibody alone).

In addition, SEM can be exploited to obtain both structural and analytical information regarding bone tissue, e.g., bone remodeling and bone pathology.^113^ In the field of bone research, the backscattered electron imaging mode is more useful than the secondary electron mode because it allows the determination of the mineral-density distribution patterns within bone.^114^ SEM can be employed to study bone resorption with the quantification of the resorption pits formed by osteoclasts in vitro.^115^ Another application is a comparison of human osteoblast growth on allografts and synthetic and xenogeneic bone grafts for bone lesion treatments.^116^

On the other hand, SEM imaging of cartilage is more challenging than that of bone due to its high water content and the need for dehydration steps during sample preparation that can lead to artifacts when analyzing the matrix structure. However, microwave fixation and cryogenic methods (e.g., freeze fracture) have been used in cartilage surface studies to overcome this limitation.^117,118^ In the work of Suso et al., environmental SEM helped obtain high-resolution images of fresh articular cartilage surfaces without sample fixation, thus minimizing the risk of creating artifacts in the structure.^119^

Overall, because of its user-friendly interface, rapid image acquisition, and ease of use, SEM is a powerful tool for imaging both materials and biological samples. The few limitations of SEM are represented by the limited size of the samples that can fit in the microscope chamber, the analyses of wet samples with conventional SEM (low vacuum and environmental SEM) and the use of conductive coating. Additionally, some solid-state X-ray detectors are not sensitive to low represented elements and cannot detect very light elements. Another limitation is the imaging of ultrafine but large structures due to the limited acquisition throughput of standard SEM.

Recently, a novel multibeam scanning electron microscope (mSEM) was developed for rapid-throughput scanning of large sample areas (e.g., a 2 mm-tissue block).^120,121^ Originally, mSEM was optimized for the quality control of semiconductor wafers at the nanometer-length scale, and afterward, in combination with sectioning and volume-rendering methods, it was used to reconstruct macroscopic volumes of murine brain tissue.^122^

Another recent advancement in imaging cellular and subcellular structures in 3D is represented by serial block face SEM (SBF-SEM) and focused ion beam SEM (FIB-SEM), more commonly used in the materials science and semiconductor fields. Although SBF-SEM can generate a stack of up to thousands of 2D images containing ultrastructural information for the bulk of the volume, it still has some limitations in terms of artifacts due to the use of a mechanical device to slice the sample and poor control of the thickness of each slide. Instead, in FIB-SEM, an ion beam is applied to carefully remove ultrathin layers of tissue, allowing for reconstruction of z-stacks capable of resolving intracellular organization in fine detail.^123^ For example, in the work of Hasegawa *et al*., the ultrastructure of cartilaginous extracellular fibrils and osteoblastic cytoplasmic processes were imaged via FIB-SEM. The results showed that osteoblasts not only extend their cytoplasmic processes to the bone matrix but also stack these cell processes on the osteoid of the primary trabeculae.^124^

There are no specific ISO procedures that apply to SEM technology; however, some ISOs should be followed to validate methods of sample processing and analysis and increase the repeatability, reliability, consistency, and overall quality of the output (Table 4). One of these documents is ISO 17025, which includes mechanisms for quality control, document control, analysis quality, and the trending of data. A more specific standard, ISO 13322-1:2014, can be applied to determine the particle size distribution of acquired SEM images. This ISO does not address the sample preparation (this is central to ISO 14887 and ISO 14488); however, a correct particle dispersion ensures accuracy of the final results.

#### Atomic force microscopy

Atomic force microscopy (AFM) belongs to the family of scanning probe microscopy (SPM) technologies and is used to image surfaces by mechanically scanning a probe over a surface and sensing the surface properties at the nanoscale or atomic scale. Specifically, a sharp probe with a nanometric tip is attached to a flexible cantilever; this probe is available with different stiffnesses and tip shapes. The cantilever deflection is recorded and provides information on the surface force. The standard geometry of the tip is a pyramid with a radius curvature of 20–30 nm.

AFM can provide not only information on the topography of the surfaces but also on mechanical, chemical, electric and magnetic properties and many others depending on the mode of operation and the property of the probe tip. Some examples of operation modes are contact mode (actual contact between the tip material and the surface), tapping mode (intermittent contact between the tip and surface, preferred for the imaging of soft biological materials), and pulsed force mode (sinusoidal movement of the cantilever for quantitative mapping of surface mechanical properties and acquisition of the surface topography in tapping mode).

AFM has been used in materials science and nanotechnology applications (e.g., imaging of polymers), biochemistry applications (nanostructural details and biomechanical properties of biomolecules, cellular components, cells or tissues), and chemistry, physics and biophysics applications.^125^ In the biological field, AFM allows us to measure the mechanical properties of the cell membrane, cell stiffness, and cell viscoelasticity and to assess cell adhesion.^126^ AFM has become an essential technique in biomedical applications,^127^ especially in the study of drug targets^128^ and has been gaining increasing interest in the field of bone and cartilage tissue engineering.

In particular, the mechanical properties of cells can be quantified from measurements of cell deformability, exploiting the AFM feature to acquire high-resolution measurements in a liquid environment.^129,130^ The resulting measure of cell elasticity (Young’s modulus) mainly reflects the deformability of the cell cytoskeleton and can be used as a marker for stem cell differentiation, as demonstrated in a study by Maloney et al.^131^. In particular, an inverse correlation between the actin fiber thickness and cell elasticity during the in vitro expansion of MSCs was demonstrated. In a recent study from Szydlak and coworkers, AFM was employed to determine the elasticity of Wharton’s jelly mesenchymal stem cells during in vitro culture.^132^ Generally, the amplitude and strength of local rupture events are not detectable by techniques performed at the level of the whole cell. Therefore, a constant velocity indentation AFM mode has been specifically used in the work from Streppa and colleagues to collect mechanical parameters of living adherent C2C12 myoblasts and myotubes.^133^

AFM is conceptually a simple technique that offers several advantages over electron microscopy, especially for imaging biological materials. This method provides a 3D surface profile at high resolutions (the z resolution is ~1 Å), does not require an expensive vacuum system and allows investigators to image the sample directly in its natural environment with minimal sample preparation (no coating, fixation) and fewer artifacts. There is generally no limitation in the medium selection, sample temperature, or chemical composition of the sample. AFM can give true atomic resolution in ultrahigh vacuum (UHV) as well as in liquid environments, and it can also be combined with a variety of optical microscopy and spectroscopy techniques, such as fluorescence microscopy or infrared spectroscopy. To avoid surface damage to biological samples, the spring constant of the cantilever must be kept at a low level (<0.2 N/m), whereas stiffer cantilevers are required to reduce noise when operating in tapping mode in air.

In contrast, one of the disadvantages of AFM is the scanning speed, which causes image distortions induced by thermal drift, followed by limitations in the scanning area (150 × 150 μm and a maximum height on the order of 10–20 μm); however, this issue can be overcome by the use of parallel probes. There is the possibility of image artifacts, which could be induced by an unsuitable tip, a poor operating environment, or scanning acquisition that is too fast. AFM images can also be affected by the nonlinearity, hysteresis, and creep of the piezoelectric material and crosstalk among the *x-*, *y-*, and *z*-axes. To overcome this crosstalk limitation, newer AFMs have introduced real-time correction software, closed loop scanners, or the use of separated orthogonal scanners.

There is a set of ISO guidelines available for scanning probe microscopy (Table 4). Most of these guidelines are of general relevance and focus, for example, the measurement of drift data (ISO 11039:2012), the determination of the cantilever normal spring (ISO 11775:2015), the vocabulary (ISO 18115:2013) and dimensional calibration (ISO 11952:2014). More detailed information can be found in the book “Quantitative Data Processing in Scanning Probe Microscopy: SPM Applications”.^134^ Additional standards, instead, are addressed to specific scanning probe microscopy techniques. For instance, ISO-27911 is specific for near-field optical scanning microscopes, and ISO 13095:2014, ISO 21222:2020 and ISO/DTR 13096 are specific for AFM. Since both the shape and the size of the AFM probe as well as the mechanism used to control the probe-sample distance can strongly affect AFM imaging, a quantitative expression of the probe tip shape is required to ensure reproducibility. Therefore, ISO 13095 specifies two methods for characterizing the shape of an AFM probe tip: a projection of the probe profile (PPP) that provides a continuous profile giving information regarding the quality of the probe, and the effective probe shape characteristic (EPSC) that provides few discrete points underlying the usefulness of a probe for depth measurements in narrow trenches. ISO 21222 describes a procedure to determine the elastic moduli of materials starting from force-distance curves acquired at the surface using AFM. The Johnson-Kendall-Roberts (JKR) two-point method is used to model the tip and surface contact in which adhesion forces outside the contact area are ignored and elastic stresses at the edge of the contact area are infinite; it applies to highly adhesive systems with low stiffnesses (elastic moduli ranging from 100 kPa to 1 GPa) and high radii of curvature.

A future improvement of high-speed atomic force microscopy is its combination with optical tweezers, which will allow the observation of a protein molecule responding to an external force applied to a specific locus in a given direction. To improve the investigation of biological samples, AFM can also be combined with standard and high-resolution fluorescence microscopy as well as Raman spectroscopy. This combination for cell imaging leads to high-resolution images that simultaneously display the 3D topography of the cytoskeleton and its stiffness and correlate them to its structural elements (e.g., distribution of actin filaments). Similarly, AFM can be coupled with a near-field scanning optical microscope to obtain simultaneous information on both the localization of molecular nanoclusters in cell membranes and protrusions. All these recent advancements are pushing AFM toward new developments in the pharmaceutical industry and clinical medicine.

### Noninvasive methods

#### Raman spectroscopy

In the forties, Raman spectroscopy (RS) became one of the most commonly used vibrational spectroscopy techniques to analyze pure chemicals. As the H_2_O Raman spectrum is very weak, RS soon developed to be a powerful tool for the analysis of biological samples.^135,136^

In the early seventies, RS was used for the characterization of native, denatured or synthetized biomolecules^137,138^ and later for the study of proteins and lysosomes from isolated animal tissues.^139^ Since then, rapid innovation in microscopy and laser technologies has made it possible to discriminate living^140^ from dead cells^141–143^ and to identify cell cycle or differentiation stages.^141,144–147^ Being label-free, chemically selective, and minimally invasive, RS rapidly caught the interest of the medical field, where it may greatly impact diagnostics or monitoring methods.

**Fig. 4:**
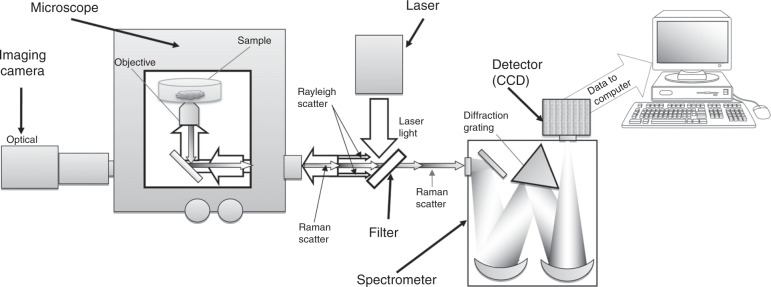
Simplified schematic of a Raman system. Reprinted by permission from Springer Nature Customer Service Center GmbH: Springer Nature. Springer eBook, Raman Micro-Spectroscopy as a Noninvasive Cell Viability Test, Verrier S. et al. © 2011^143^

**Fig. 5:**
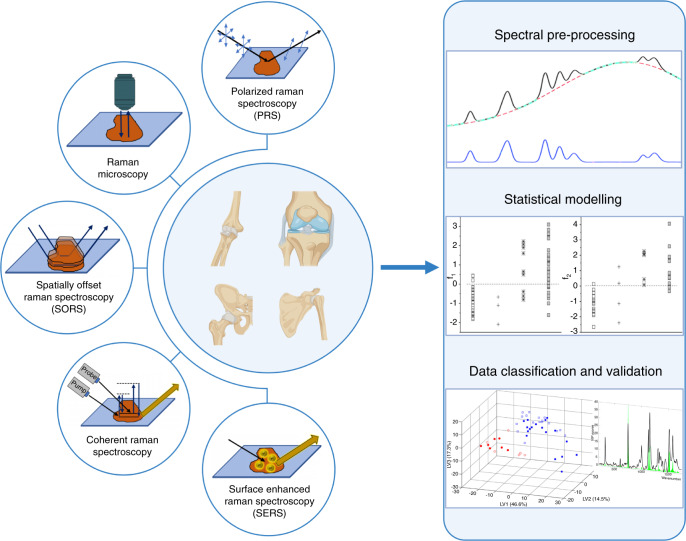
Summary of Raman spectroscopy techniques: polarized Raman spectroscopy (PRS), suitable for highly oriented systems; Raman microscopy, able to couple chemical and morphological information; spatially offset Raman spectroscopy (SORS), used for collecting Raman signal from deep regions; coherent Raman spectroscopy (SRS & CARS), able to improve Raman signal intensity up to 10^5^ factor; and surface enhanced Raman spectroscopy, (SERS) able to enhance signal intensity up to 10^10^ factor, exploiting the plasmonic effect between the sample and metal particles. The processing of Raman data collected from biological samples is accomplished by spectral analysis and statistical treatments

A basic RS setup (Fig. 4) comprises a laser source typically in the near infrared range, a series of mirrors and a microscope directing the light toward the sample. After interaction with the sample, only a small portion of the scattered photons change their frequency. Following the filtration of nonshifted photons, photons with modified frequency are directed toward a spectrometer linked to a detector system (CCD), from which the data are transferred to a computer. However, as the Raman signal is very weak, in recent decades substantial effort has been devoted to finding ways to increase it.^136^ To date, there have been more than twenty enhanced RS techniques characterized by an amplified signal, and here, some of them are summarized in Fig. 5.

In spontaneous RS, the Stokes signal is detected under continuous radiation, typically from a diode laser with high spectral stability. For in vivo clinical RS applications, it is most convenient to deliver light to a tissue and collect it from the tissue by means of specifically designed fiber probes.^148,149^ However, one of the largest challenges of fiber probe design is to minimize tissue fluorescence.

Confocal RS implements a confocal microscope configuration to provide optical depth sectioning by spatial filtering of the collected RS light with a pinhole or an optical fiber to block out-of-focus signals. To date, confocal Raman probes have been used mainly for ex vivo and in vitro studies.^150,151^

Spatially offset RS (SORS) is similar to RS but collects Raman signals from deeper regions of the tissue by spatially offsetting the detection and excitation fibers. Collecting Raman signals at different offsets effectively samples different layers in the tissue. SORS typically uses a probe with an illumination fiber surrounded by detections fibers offset of 1–5 mm,^152,153^ but an offset as high as 16 mm has been used to perform Raman tomographic imaging in bone.^154^

Coherent RS uses two light fields (referred to as the pump and Stokes beam) so that the difference corresponds to a vibrational mode frequency of a molecular bond of interest. The coherent addition of the Raman signal from different molecules improves the signal compared to spontaneous Raman, typically by up to ~10^5^.^155^ Coherent RS techniques include stimulated RS (SRS) and coherent anti-Stokes RS (CARS). Both SRS and CARS can be performed in highly fluorescent media, which is usually a rather limiting factor for Raman imaging in tissues.

Polarized RS (PRS) provides information regarding both the chemical composition and anisotropic directions of highly oriented systems, such as the amide I band and alpha helical conformation, representative of collagen.^156^

In the field of regenerative medicine and tissue engineering, Dooley et al.^157^ used phantom samples based on 3D-printed polycaprolactone (PCL):HA scaffolds to investigate the feasibility of SORS for monitoring the mineralization of bone tissue engineering scaffolds in large animal models. It was demonstrated that SORS was able to detect HA concentrations that were an order of magnitude lower than those found in living bones, even when a 4 mm thick layer of skin (mimicking in vivo transcutaneous measurements) was present.

Liao et al.^158^ disclosed the possibility of applying SORS for nondestructive analysis of bone tissue engineering scaffolds. A multilayered scaffold composed of bioactive glass foams (IEIC16), a 3D-printed biodegradable poly(lactic-coglycolic acid) scaffold and HA powder was used to model 3D tissue engineering constructs at a real scale for nondestructive investigation of the biomineralization process. The authors were able to evaluate spectral depth profiles at high speed (5 s for each spectrum) and the simplicity of SORS application as a promising noninvasive approach to study cell/tissue growth and for in vitro and in vivo monitoring of the 3D scaffold long-lasting biomineralization process.

With regard to Raman databases and standards, to date, available and free Raman spectra databases can be found only for minerals, inorganic materials, or simple organic molecules. For example, Bio–Rad’s SpectraBase (https://spectrabase.com) has over 24 000 spectra available, although most are basic organic compounds. Unfortunately, comprehensive databases of Raman spectra of biological compounds and tissues are still not available due to the complexity of biological systems and the necessity of standardization of Raman data. Nevertheless, some review articles reported an extensive collection of the most relevant Raman bands that can be found in a Raman tissue investigation. The review of Talari et al.^159^ represents today the most complete list of assigned Raman peaks from biological specimens. Talari reported in total more than 1 000 assigned bands extrapolated from Raman spectra belonging to both healthy and pathological tissues.

To meet the requirements of standardization, multidisciplinary and multicenter networks, such as Raman4Clinics (EU COST Action BM1401) and the International Society for Clinical Spectroscopy (ClirSpec), have recently been established to compare 35 Raman spectroscopic instruments and setups in 7 European countries.^160^

In addition, in a very recent study, Power et al. first proposed RS as a quality control tool for GMP-compliant manufacturing of tissue engineered cartilage.^161^ Current GMP-compliant quality controls for tissue engineering procedures include sterility testing and required cell number and viability testing. In addition to the current requirements, the authors propose to use RS to assess the quality of the donor site tissue (e.g., purity) and of the resulting tissue engineered construct prior to implantation.

RS analysis of biological samples contains much information, but once measurement procedures and data analysis protocols have been standardized and validated with other classical methods, Raman spectra can be produced and analyzed in a quick, automated, and noninvasive manner.

#### Microcomputed tomography

**Fig. 6:**
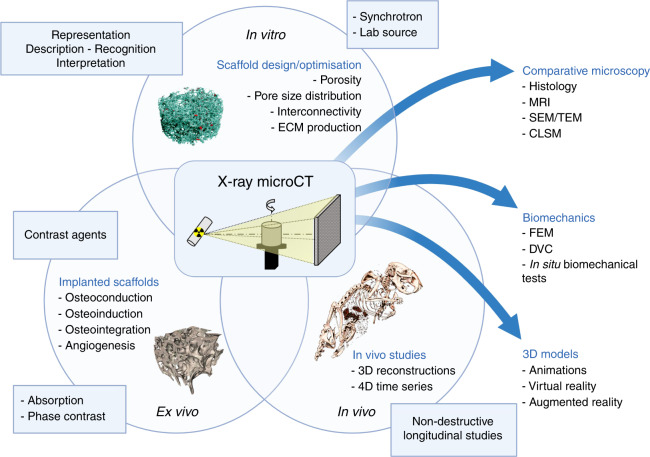
Schematic representation of the potential of microCT analysis for musculoskeletal tissue engineering. This nondestructive technique can be used for different applications for biomaterials per se or in combination with cells and tissue (both ex vivo and in vivo). The produced image dataset can be compared with that from different microscopy techniques, combined with biomechanical tests or used for 3D modeling

The application of imaging techniques based on microcomputed tomography (microCT) in the field of tissue engineering has been steadily increasing in importance for over a decade (Fig. 6). MicroCT can be used in different contexts (in vitro, in vivo and ex vivo) for the 3D and even 4D qualitative and quantitative evaluation of engineered materials, both in terms of optimization of the design and measurement of their regenerative capacity.

The technique is nondestructive and is based on the interaction of X-rays with matter. A series of radiographic projections taken at different angles is captured by a detector generating bidimensional images. Using computer algorithms, these projections are used to reconstruct the internal and external structure of the sample.^162,163^

The most common method of scanning is based on the attenuation of X-rays when they pass through matter. This makes the technique largely used for studying many aspects related to bone biology. In the field of tissue engineering, a wide range of materials can be directly examined, such as metals, ceramics, some polymers and mineralized tissues.^162^ The study of the morphological characteristics that are closely related to certain quality requirements, such as their regenerative properties, is crucial for improving existing materials or creating new products.^164,165^ In this context, the ability to obtain 3D datasets using microCT provides more accurate information on the structure of the sample than complementary two-dimensional (2D) methods, such as histology and scanning electron microscopy.^166,167^ Therefore, microCT is commonly used in the morphological and structural analysis of bone tissue engineering scaffolds prior to cell culture or implantation^168–170^ and in the in vitro evaluation of the influence of porosity on cell seeding.^171^ The nondestructive quantification of characteristics such as porosity, pore size distribution and interconnectivity is a key factor in terms of planning and optimization of scaffold designs because a defined porosity allows cell migration, improves proliferation and production of extracellular matrices and facilitates tissue growth and the invasion of blood vessels.^172,173^ Moreover, microCT is widely used in the evaluation of the extracellular matrix growth process within scaffolds.^174^ MicroCT provides a noninvasive and nondestructive tool for the evaluation of mineralization based on in vitro culture conditions,^175–177^ the osteoinductive and osteoconductive properties of scaffolds through the 3D quantification of newly formed bone in preclinical models ex vivo^178–180^ or longitudinally in vivo,^181,182^ and the resorption and degradation of biomaterials in vitro and in vivo.^183–185^

The morphological analysis of scaffolds can be coupled with mechanical tests (e.g., porosity versus mechanical load resistance), providing an additional rational basis for the design and optimization of scaffolds. For example, microCT imaging can act as a source for finite element (FE) analysis, increasing the simulation modeling accuracy,^186^ and it can be used for the dynamic monitoring of compressive strength.^187,188^ In addition, the combination of microCT imaging and mechanical tests allows a digital volume correlation (DVC) approach to study the micromechanics of bone-biomaterial systems.^189^

Although microCT is mainly used for mineralized tissues, currently the imaging of materials or tissues with a lower attenuation coefficient, such as hydrogels, natural polymers, cells, and soft tissues, is possible due to the increasingly widespread implementation of contrast agents.^166,167^ For example, osmium tetroxide (OsO_4_) has been used to increase the contrast of some polymers scanned in an aqueous medium^166,167^ or to evaluate 3D cell colonization inside scaffolds in combination with DNA quantification.^190,191^ Mixtures of osmium tetroxide and uranyl acetate or uranyl acetate and lead citrate were used for the study of collagen-based scaffolds.^192^ Iodine-based or phosphotungstic acid (PTA) contrast agents have been used for in vitro extracellular matrix visualization inside engineered scaffolds.^193,194^

However, these contrast agents are often toxic, making longitudinal and nondestructive evaluation impossible.

In addition to absorption, it is possible to use approaches based on other aspects of the X-ray/matter interaction. X-ray imaging techniques based on phase contrast (PC) use X-ray refraction and phase shifting. PC microCT has great potential in biomedical applications because variations in refractive indices are generally higher than variations in X-ray attenuation coefficients. Therefore, information on the structure of soft tissues or materials with low absorption can be theoretically obtained without contrast agents.^195,196^ PC techniques have been applied in the field of tissue engineering for the visualization of polymeric scaffolds under cell culture conditions,^197,198^ for the characterization of polydimethylsiloxane scaffold deformation caused by ultrasounds,^199^ and for the detection of 3D cell organization in polyglycolic acid—polylactic acid (PLGA) scaffolds.^200^

MicroCT images can also be used as valid input for 3D bioprinting, improving the manufacturing of increasingly complex and customized scaffolds.^201^

The advantages of the microCT imaging technique can therefore be summarized in its noninvasive and nondestructive intrinsic characteristics, the possibility of high-resolution 3D visualization, the possibility to perform longitudinal studies on the same sample, and digital-based information that can be used to mathematically describe a 3D object. MicroCT can be used both to integrate existing analysis techniques and in combination with them because the analyzed samples can then be histologically assessed or biomechanically tested.^202^ In addition, continuous hardware and software innovations increase the 3D rendering capabilities up to virtual reality and augmented reality applications.

The main limitations of the technique lie in the significant effect of acquisition settings (e.g., nominal resolution and the rotation step) on image quality,^203^ in the difficult identification of correct thresholding values for materials or tissues with similar absorption coefficients,^183^ and in the radiation dose in in vivo longitudinal studies.^204,205^ In addition, microCT imaging acquisition and analysis are not standardized. However, the coordination of the results can be maximized using references that describe the validation of the methods through a comparison and correlation with other traditional techniques such as histomorphometry.^206,207^

In the context of good laboratory practice (GLP) compliance, specific applications can be validated through SOPs, which mainly take into account the generation, integrity and monitoring of electronic data, calibration of the systems and validation of associated software. Nevertheless, nonclinical imaging data can be incorporated into regulatory communications even if they are not GLP compliant. In this respect, it is preferable to describe in detail (1) the imaging procedure enclosing complete descriptions of hardware and software; (2) the degree of reliability, quality, and integrity of the electronic data to be able to track and reconstruct the data processing; and (3) the data management and archiving procedures. Measures to prevent unintentional image changes and backup and restore procedures should also be considered^208^.

Some international standards in the field of computed tomography have been published, but none cover the validation and calibration of CT systems. The most important published standards for CT are summarized in Table 5.

**Table 5:** Published standards applicable to computed tomography (CT)

Standard code	Standard title	Brief description of document
ISO 15708-1:2017	Nondestructive testing - Radiation methods for computed tomography - Part 1: Terminology	Describes the terms used in the field of computed tomography (CT)
ISO 15708-2:2017	Nondestructive testing - Radiation methods for computed tomography - Part 2: Principles, equipment and samples	Describes the general principles of X -ray CT
ISO 15708-3:2017	Nondestructive testing - Radiation methods for computed tomography - Part 3: Operation and interpretation	Provides technical information to enable the selection of suitable data acquisition and image reconstruction parameters for the interpretation of results
ISO 15708-4:2017	Nondestructive testing - Radiation methods for computed tomography - Part 4: Qualification	Provides a set of CT performance parameter definitions and their relation to CT system specification
ASTM E1441-19	Standard Guide for Computed Tomography (CT)	Describes the general principles of X -ray CT
ASTM E1570-19	Standard Practice for Fan Beam Computed Tomographic (CT) Examination	Establishes the minimum requirements for computed tomography (CT) examination
ASTM E1672-12	Standard Guide for Computed Tomography (CT) System Selection	Provides a common terminology to guide both purchaser and supplier in the CT system selection process
ASTM E1695-95 (Reapproved 2013)	Standard Test Method for Measurement of Computed Tomography (CT) System Performance	Describes the method to determine the spatial resolution and the contrast sensitivity of an X-ray CT system

#### Optical coherence tomography

A technology to overcome the label-dependent nature of optical tissue microscopy is optical coherence tomography/microscopy (OCT/OCM). This is an imaging technique with a resolution of >10 µm and penetration depths of up to 2 mm. In OCT, the reflected light of a broadband, low-coherence light source is detected by an interferometer, which results in clear 3D images. Despite its limitations in resolution, it can display macrostructural tissue morphologies with a label-free method and is even compatible with calcified scaffolds,^209–211^ making it particularly relevant for monitoring cell proliferation,^212,213^ musculoskeletal structures^209^ and diseases.^210^

Special modes of OCT can disclose more information with respect to a tissue construct than only its still complex morphology. Optical coherence elastography (OCE), for instance, essentially images a tissue undergoing mechanical deformation.^214^ Local strain distributions are then estimated based on cross-sectional maps of the mechanically induced (cell) displacement. OCE has thus the potential to mechanically characterize engineered tissue, essentially contributing to final quality control. However, more than the tissue itself can be analyzed by different modes of OCT; for example, Ghosn et al. reported the use of OCT for label-free imaging of glucose diffusion within tissues, thereby underpinning the versatility and potential of OCT for analytical tissue engineering imaging.^215^

### Conservative methods

#### Electrochemical sensors and biosensors

**Fig. 7:**
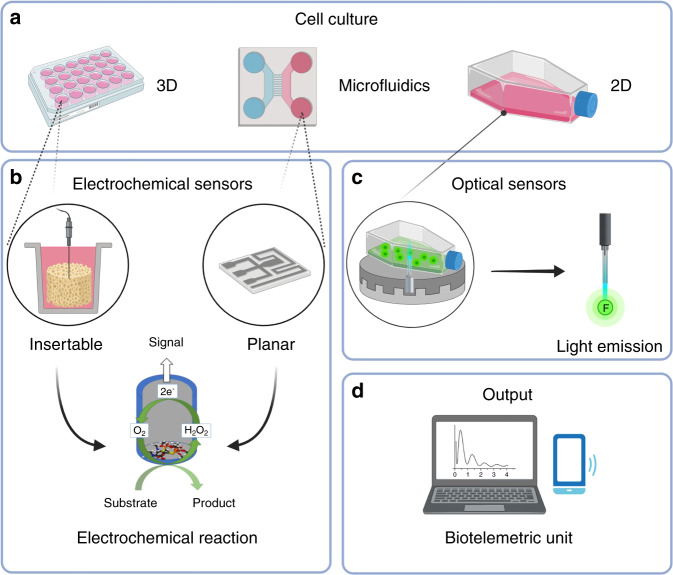
Overview of the applicability of sensors to the field of tissue engineering. Critical parameters for construct quality control (e.g., glucose, lactate, and oxygen consumption) could be monitored in different types of in vitro culture systems (**a**) by using electrochemical (**b**) and optical (**c**) sensors/biosensors for real-time quality monitoring (**d**). These methods are conservative, allowing for longitudinal studies and further applicability of tissue engineered constructs

In disciplines such as neurosciences, for more than a decade, sophisticated analytical monitoring techniques have been in use for the in vitro and in vivo monitoring of chemical processes in real time. The use of these techniques is still limited in the fields that are the focus of this review. However, it is envisaged that the use of sensors, particularly miniaturized microsensors, can be developed, implemented, and adapted ad hoc for the study and monitoring of the cellular quality of 3D constructs for musculoskeletal tissue engineering and biofabrication (Fig. 7). From the technological, qualitative, and economic point of view, the translation of expertise and sensors from neuroscience to in vitro applications and tissue engineering would bring quality control of implants to the next level and thus accelerate translation to clinical applications.

During the last few decades, biosensing technology has attracted much attention due to the numerous features that make it applicable in several fields, such as environmental, agri-food, and biomedical, both in vivo and in vitro.^216–220^ Sensors and biosensors are widespread not only in diverse fields of application but also because they offer the possibility of being miniaturized. This characteristic allows real-time monitoring of several compounds (e.g., glucose, lactate, glutamate) at very low concentrations depending on the sensor used, thus representing a noninvasive measurement for direct analysis of dissolved molecules. In addition, the low production cost in manufacturing makes these biosensors particularly appealing, as they are economically sustainable for researchers. Indeed, in recent years, electrochemical sensors and biosensors have become interesting tools for application in tissue engineering and regenerative medicine.^216–221^

The characteristics of an electrochemical sensor can be modified for interaction with one or more analytes, transforming the obtained chemical signal into a quantifiable electrical signal. Depending on the electrochemical technique used (voltammetry, amperometry, conductometry or potentiometry), this signal can be linearly or logarithmically proportional to the analyte concentration. Amperometric microsensors are valued devices for both in vivo and in vitro detection of different compounds, such as dopamine,^222–224^ ascorbic acid,^222,225,226^ nitric oxide (NO)^227^, and oxygen (O_2_).^225,228^ In addition to the high sensitivity and quite high spatial and temporal resolution, microsensors are very interesting because they allow the real-time monitoring of the compounds under examination.

**Fig. 8:**
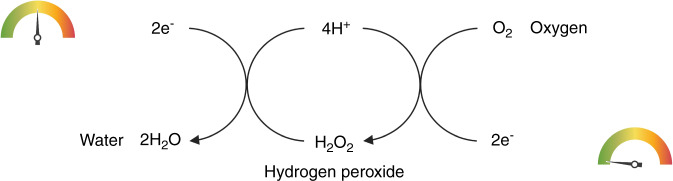
The two-step reduction of oxygen allows the formation of hydrogen peroxide as a detectable intermediate

Amperometric devices can work in both oxidation and reduction modes. Among the monitored reducible compounds, O_2_ is one of the most important because of its physiological and biochemical implications. Oxygen monitoring occurs when a cathodic potential is used.^225,228,229^ O_2_ reduction arises by means of a two-step reaction, leading to H_2_O production (Fig. 8).

A biosensor is used when the compounds dissolved in aqueous matrices cannot be directly oxidized or reduced on the surface of the transducer, as in the case of glucose, lactate, glutamate, ethanol, or polyphenols.^219,220,230,231^ The term biosensor indicates a chemical sensor in which the recognition system uses a biochemical or biological sensing element. Depending on the technology, biosensors can be divided into two main categories: biocatalytic devices, where enzymes, tissues or cells are used, and bioaffinity sensors, when antibodies, nucleic acids, or receptors are employed. In particular, amperometric biosensors have become very attractive because of their sensitivity, fast response, and high spatial resolution. The first amperometric sensor was developed for the measurement of oxygen tension.^232^ Since then, amperometric sensors have evolved and have become important tools in research due to their numerous technical advantages. In neuroscience, biosensors have been integrated into telemetry systems, allowing direct signal transmission and the simultaneous monitoring of different analytes. In the biomedical field, biosensors are commonly employed for the real-time monitoring of different important compounds, such as neurotransmitters (e.g., glutamate),^233–235^ glucose and lactate.^235–237^

**Fig. 9:**
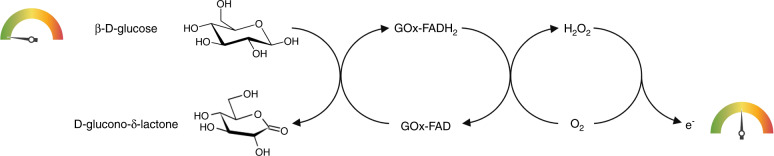
The enzymatic oxidation of glucose produces hydrogen peroxide whose concentration is directly proportional to that of glucose

The most common amperometric biosensors are enzyme-based. These devices exploit the capability of some enzymes of the oxidase class to convert the compound of interest into an amperometrically detectable analyte. Indeed, in a biosensor for glucose, glucose oxidase (GOx) allows its conversion in the presence of oxygen, as depicted in Fig. 9. The concentration of H_2_O_2_ produced from the GOx reaction is directly proportional to the glucose concentration in the matrix, and H_2_O_2_ is easily oxidized on a platinum surface when an anodic potential is applied.^220,230,231,238^

Enzyme-based biosensors, based on platinum wire modifications, have proven to be particularly useful when implanted in brain tissues in preclinical models due to their biocompatibility and to their high spatial and temporal resolution. Depending on the application, such as the study of the cellular microenvironment in 2D cell cultures,^239,240^ sensors and biosensors can be developed with different shapes, such as cylindrical and conical. The main advantage is related to the small dimensions (50–500 μm), which allow them to be inserted inside tissues and organs. Planar sensors have been developed as well^241,242^ since these types of sensors and biosensors also offer a notable detection capability, simplicity, and low cost.

Although the application of biosensors in tissue engineering and regenerative medicine is still limited, these tools can bring great possibilities in the field.^221^ Through these tools and these techniques, it is possible to check many critical parameters to ensure proper tissue growth,^243^ such as oxygen and nutrient uptake or the release of metabolites. In addition, monitoring metabolic parameters can contribute to the standardization of experiments. Sensor-based systems for cell metabolism monitoring have been recently reviewed by Kieninger and coworkers.^240^ The investigation showed an overview of the possibility of using sensor systems to track cell metabolism even in 3D cultures. It is possible to directly measure extracellular substances in the medium (e.g., glucose, lactate, oxygen),^244,245^ but matrix interference is an important issue to address^217^ to make the monitoring of biosensors reliable and reproducible. Several studies have been performed by means of integrated microfluidics techniques^246,247^ and through modification of flasks and plates. Compared to batch systems, microfluidics techniques allow important advantages, such as dimensions or sample dilution.^248^

The materials used for sensor construction need to be biocompatible, nontoxic and sterilizable. Different sterilization strategies can be successfully followed,^249^ especially in combination, but any procedure has to be adapted to each sensor depending on the design chosen. Accordingly, inactivation and loss of the recognition element must be considered, particularly with regard to enzyme-based biosensors. However, several development approaches, such as enzyme immobilization or biosensor storage, can be used to maintain and improve the analytical performances of these tools.^217^

Future prospectives on amperometric microsensors and biosensors are focused on the development of nanostructured transducers because of new and improved functionalities that cover a wide variety of applications in the biomedical field and on the improvement of the duration of analytical performance in terms of enzyme activity.

#### Optical tissue environment monitoring

An easy and nondestructive way to assess cell metabolic activity or physiology is the analysis of the cell/tissue environment, namely, the cell culture medium. Sampling of small volumes of the medium allows the quantification of a multitude of metabolites and nutrients; typically, even less than 5 µL is sufficient for the analysis of glucose, cholesterol, glutamate, lactate dehydrogenase, ions, or total protein content, among others.^250^ Such assay-based kits are available in small size for use in multiwell plates with optical plate readers or even at industrial scale-throughput packs to be used in conjunction with specialized spectrometers. While metabolite quantification is not a new concept, it is still not widely used in tissue engineering applications. However, as process analytical technologies (PAT) originating from the field of pharmaceuticals (U.S. Food and Drug Administration. Guidance for Industry: PAT—A Framework for Innovative Pharmaceutical Development, Manufacturing, and Quality Assurance) become more relevant for transplantation of engineered tissue constructs, they in turn will await their standard employment in modern tissue engineering.

Such in-depth quantifications of cell/tissue environmental parameters are already well-established solutions – as long as enough liquid is available for sampling. When the total amount of available liquid does not allow for sampling or sampling is technically not feasible, optical probes are available for a small range of cell environmental parameter analyses. These probes are typically based on parameter-sensitive fluorescent dyes: for instance, a fluorophore that changes its fluorescence intensity and fluorescent lifetime upon a change in pH or oxygen concentration. Examples of fluorophores are ruthenium- and metallo-porphyrin-based molecules. Due to their cytotoxic nature, they are typically encapsulated into polymers, sol-gels, or silica matrices. The encapsulated formats can be beads, rods, flat pads, and other geometrical objects to either flow through an entire fluidic system or be located at a region of interest. Since the parameter sensitivity of the currently available fluorophores is still limited, most commercially available systems offer probes for pH and oxygen only. Nevertheless, pH and oxygen are two essential parameters for the development of tissue engineering modules and reactors, which could benefit from the implementation of probes that pose a good alternative to electrochemical sensors. The implementation does, however, have direct implications on the module/reactor design, as the probes must be optically accessible and the optical penetration depth into a tissue construct is very limited. Given that the implementation does not affect the cell/tissue culture and a USP class-VI polymer is chosen for fluorophore encapsulation, these optical probes can pose a noncytotoxic sensor for tissue constructs engineered for transplantation.

## Summary and prospectives

The main purpose of tissue engineering is to create functional and implantable constructs able to restore damaged or lost tissue. In recent years, while the acceleration of technological development has fostered great improvements in the field, the translation of tissue engineered constructs into the clinic is still limited. In the biological field, a variety of methods have been proposed to improve the reproducibility of protocols in terms of cell isolation and differentiation toward targeted tissues. The process of cell embedding and maturation in biomaterials has also been developed further to move toward functional cell-laden implants. With the recent advances of technologies, skills, and methodologies to produce complex constructs that mimic the composition, macrostructure or microstructure of native tissues has been developed. However, from laboratory to clinical practice, the gap is still vast. One of the main challenges limiting the clinical translation of tissue engineered constructs is the lack of quality control of the end construct. Indeed, if the chemical, physical and mechanical properties of a biomaterial are reproducible, then the cellular component of such constructs is still subject to interindividual variations or cell manipulation that could hinder the cell-scaffold interaction, and therefore, the overall properties and potentially the efficiency of the construct can be affected. Another major lacking aspect regarding cell viability and cell maturation is the possibility of monitoring 3D constructs during in vitro culture. A real-time, noninvasive monitoring system would be of paramount importance to assess key biological parameters and possibly control cell differentiation processes, matrix production, cell-scaffold interactions, and overall development of the construct.

To date, there is no single and dogmatic assay to study the quality of 2D and 3D constructs. However, depending on need and accessibility, one technique can be chosen over another. The major limitation of the techniques routinely used in tissue engineering/regenerative medicine is that they often involve sample destruction. Due to the invasive character of these techniques, the samples cannot be used for further analyses; therefore, multiple constructs are needed to collect all required information.

In contrast, methods defined as noninvasive, such as confocal or microCT, allow preservation of the sample during the analysis, but they might subject the sample to strong stress, which could then invalidate the final quality. In recent years, further methods, e.g., Raman spectroscopy, have been developed and translated to biological and medical analysis. Though the current lack of RS standardization is the largest challenge for its translation in the clinic, no particular sample preparation is required, and Raman measurements can be carried out both in vitro and in vivo (i.e., intraoperative) conditions.

There is a clear need for the development or translation of noninvasive methodologies to the field of regenerative medicine and tissue engineering to bring the field one step closer to its clinical application. Currently, conservative methods such as minimally invasive sensors for musculoskeletal tissue engineering are completely unexplored but are envisaged to be determinant for the translation of solutions from the preclinical stage. Sensors also have great potential to facilitate the initial optimization of constructs and could be integrated into a more complex workflow that comprises the analysis of different parameters using multiple techniques and possibly allows the monitoring of the same sample over time (at least with some combinations). This “multidimensional” system for the quality control of constructs is highly advisable to exploit the advantages and overcome the limitations of single techniques. For example, above all the advantages of sensors, their main drawback is that they do not provide morphological data, which can in turn be retrieved by combination with other techniques.

As discussed in this review, recent technologies show promising advancements. The implementation of different sets of monitoring technologies will nevertheless remain the only solution for quite a while since the complexity of tissue engineering aspects imposes a plethora of physiologically relevant functions that cannot be assessed using a single technology at hand today. Thus, the combination of different advanced techniques and progress in laboratory applications/representations will improve the quality in the field of tissue engineering toward complete customization of the constructs and increased effectiveness of personalized medicine strategies. In the future, the choice of noninvasive strategies will furthermore outmatch invasive handling due to the limited availability of patient-derived cells as well as its potential to monitor tissue development repeatedly over time, providing insights into crucial morphological development.
