# Author Correction: Quality control methods in musculoskeletal tissue engineering: from imaging to biosensors

**DOI:** 10.1038/s41413-021-00174-w

**Published:** 2021-12-28

**Authors:** Daniele Zuncheddu, Elena Della Bella, Andrea Schwab, Dalila Petta, Gaia Rocchitta, Silvia Generelli, Felix Kurth, Annapaola Parrilli, Sophie Verrier, Julietta V. Rau, Marco Fosca, Margherita Maioli, Pier Andrea Serra, Mauro Alini, Heinz Redl, Sibylle Grad, Valentina Basoli

**Affiliations:** 1 AO Research Institute Davos, Clavadelerstrasse 8, 7270 Davos, Switzerland; 2 Regenerative Medicine Technologies Lab, Ente Ospedaliero Cantonale (EOC), Via Tesserete 46, 6900 Lugano, Switzerland; 3 Department of Medical, Surgical and Experimental Sciences, University of Sassari, Viale San Pietro 43/b, 07100 Sassari, Italy; 4 Centre Suisse d’Electronique et de Microtechnique, Bahnhofstrasse 1, 7302 Landquart, Switzerland; 5 Center for X-ray Analytics, Empa—Swiss Federal Laboratories for Materials Science and Technology, Überlandstrasse 129, 8600 Dübendorf, Switzerland; 6 Istituto di Struttura della Materia, Consiglio Nazionale delle Ricerche (ISM-CNR), Via del Fosso del Cavaliere, 100 - 00133 Rome, Italy; 7 Sechenov First Moscow State Medical University, Trubetskaya 8, build. 2, 119991 Moscow, Russian Federation; 8 Department of Biomedical Sciences, University of Sassari, Viale San Pietro 43/b, 07100 Sassari, Italy; 9 Ludwig Boltzmann Institute for Experimental and Clinical Traumatology in AUVA trauma research center, Donaueschingenstraße 13, 1200 Vienna, Austria; 10 Austrian Cluster for Tissue Regeneration, Vienna, Austria

**Keywords:** Bone, Bone quality and biomechanics

Correction to: *Bone Research* 10.1038/s41413-021-00167-9, published online 27 October 2021

Following publication of this article [1], the authors would like to change the order of the affiliations 6 and 7. The correct order is below.

6 Istituto di Struttura della Materia, Consiglio Nazionale delle Ricerche (ISM-CNR), Via del Fosso del Cavaliere, 100 - 00133 Rome, Italy;

7 Sechenov First Moscow State Medical University, Trubetskaya 8, build. 2, 119991 Moscow, Russian Federation;

Both authors Julietta V. Rau and Marco Fosca are affiliated with affiliation number 6, and only Julietta V. Rau is affiliated with affiliation number 7.

The original article has been updated.

## References

[1] Zuncheddu D, Della Bella E, Schwab A (2021). Quality control methods in musculoskeletal tissue engineering: from imaging to biosensors. Bone Res..

